# Guided Alveolar Ridge Preservation (G-ARP) with a Subperiosteal Cortical Lamina: A Retrospective Observational Case Series

**DOI:** 10.3390/dj14060361

**Published:** 2026-06-11

**Authors:** Orlando Guerra Cobian, Giacomo Mainetti, Franco Bengazi, Tomaso Mainetti, Karol Alí Apaza Alccayhuaman, Mie Nonaka, Daniele Botticelli

**Affiliations:** 1 Department Oral Surgery, Faculty of Dentistry, University of Medical Science of La Habana, La Habana 10400, Cuba; guerracobian@gmail.com (O.G.C.); f.bengazi@virgilio.it (F.B.); 2 ARDEC Academy, 47923 Rimini, Italy; giacomo.mainetti@hotmail.it (G.M.); tmainetti@libero.it (T.M.); karol.ali.apazaa@gmail.com (K.A.A.A.); mail@nonaka-dc.com (M.N.); 3 Biomaterials and Technology, Department Research, University Center for Dental Medicine Basel UZB, University of Basel, 4058 Basel, Switzerland; 4 Nonaka Dental Clinic, Kobe Clinic Building 3F, 3-1-7 Nunobiki-cho, Kobe 651-0097, Hyogo, Japan

**Keywords:** alveolar process, bone regeneration, cone-beam computed tomography, guided tissue regeneration, retrospective studies, tooth extraction, periosteum

## Abstract

**Background/Objectives:** This retrospective study evaluated the dimensional behavior of an alveolar ridge preservation approach based on the placement of a subperiosteal heterologous cortical collagenic bone lamina without the use of particulate grafting material under routine clinical conditions. **Methods:** Seventy consecutive patients, each contributing one extraction site, were included. All sites were treated with a cortical lamina placed on the buccal aspect of the socket immediately after tooth extraction. Cone-beam computed tomography scans were obtained before extraction and after approximately 6 months of healing. Crestal width and vertical dimensions at the buccal, central, and lingual aspects were measured. An exploratory subgroup analysis was also performed according to the tooth region. **Results:** Over a mean follow-up period of 6.1 ± 1.4 months, the mean crestal width decreased from 8.4 ± 2.8 mm to 7.9 ± 1.8 mm, corresponding to a mean reduction of −0.6 ± 2.0 mm (*p* = 0.001). The central vertical measurement showed no significant change (0.4 ± 2.6 mm; *p* = 0.531). Buccal height remained stable, whereas lingual height showed a small reduction (−0.6 ± 1.7 mm; *p* = 0.005). The exploratory subgroup analysis revealed a width change of −0.3 ± 1.6 mm for anterior sites and 0.1 ± 2.0 mm for premolar sites while a more pronounced reduction in ridge dimensions was observed at molar sites (−2.2 ± 1.6 mm). **Conclusions:** The cortical lamina approach was associated with limited post-extraction dimensional changes and may represent a useful option for alveolar ridge preservation without the use of particulate grafting material, although less favorable dimensional stability may be expected in molar regions.

## 1. Introduction

Modern implant dentistry increasingly relies on precise control of peri-implant hard and soft tissue volumes to achieve predictable functional stability and long-term esthetic outcomes. Following tooth extraction, pronounced remodeling of the alveolar process occurs as a physiological consequence of socket healing and the loss of tooth-dependent periodontal structures, often resulting in marked dimensional alterations of the ridge that may complicate subsequent implant placement and prosthetic reconstruction [1,2,3].

To counteract this unfavorable biological cascade, several alveolar ridge preservation (ARP) strategies have been proposed, most of which are based on the placement of grafting materials within the extraction socket, either alone or in combination with barrier membranes. Although these approaches have generally been shown to reduce post-extraction ridge resorption, systematic reviews and meta-analyses have reported considerable heterogeneity in outcomes, likely reflecting differences in biomaterials, surgical protocols, socket and defect morphology, and healing dynamics [4,5,6,7]. In addition, concerns remain regarding delayed graft remodeling, persistence of residual biomaterial, and incomplete maturation of regenerated bone in some grafted sites [8,9].

In this context, a novel approach has been introduced, consisting of the subperiosteal placement of a rigid barrier, such as a high-density polytetrafluoroethylene (d-PTFE) membrane or a cortical lamina, between the elevated flap and the buccal cortical plate immediately after tooth extraction, with some studies combining this procedure with grafting material and others adopting a particulate graft-free protocol [10,11,12,13,14,15,16,17]. The biological mechanisms potentially underlying the effectiveness of this technique are still debated and may involve either periosteal isolation, with a consequent reduction in osteoclastic activity, or the local application of guided bone regeneration principles during socket healing.

Recent experimental observations on socket entrance healing provide additional biological support for an alveolar ridge preservation strategy performed without particulate grafting material. In one study, ARP performed with a partially resorbing collagenic xenograft resulted in incomplete corticalization of the socket entrance, with residual graft particles remaining in the most coronal portion of the newly formed alveolar bone [18]. Because this region lies directly beneath the mucosal seal, it may be particularly relevant to the biological stability of the future peri-implant interface. In the same experiment, however, the use of a more resorbable grafting material was associated with almost complete replacement by newly formed bone and with a corticalization pattern at the socket entrance comparable to that observed in ungrafted sites [18,19].

On the basis of this biological rationale, together with the encouraging results previously reported with cortical lamina-based approaches, a pilot randomized controlled trial preliminarily evaluated the clinical effectiveness of a lamina-based alveolar ridge preservation protocol performed without particulate grafting material [20]. That study showed significant preservation of alveolar ridge dimensions together with favorable bone healing in the absence of particulate grafting materials. However, although randomized trials provide controlled clinical evidence, their external validity may be limited by strict inclusion criteria, standardized surgical conditions, and highly selected patient populations.

By contrast, routine clinical practice involves post-extraction defects with greater anatomical variability, heterogeneous healing potential, and a broader range of local and systemic factors that may influence treatment outcomes. Therefore, evaluating the reproducibility, robustness, and clinical performance of this protocol under real-world conditions is essential to better define its actual therapeutic value. Accordingly, the aim of the present retrospective study was to assess the clinical effectiveness of a subperiosteal heterologous cortical collagenic bone lamina approach for alveolar ridge preservation in a consecutive series of patients treated in daily clinical practice. The primary objective was to evaluate horizontal and vertical dimensional changes in the alveolar ridge following tooth extraction and treatment with the subperiosteal cortical lamina approach under routine clinical conditions.

## 2. Materials and Methods

### 2.1. Ethical Statements and Study Design

This single-center retrospective observational clinical study was designed to assess dimensional changes in the healed alveolar ridge following a Guided Alveolar Ridge Preservation (G-ARP) procedure based on the application of a heterologous cortical collagenic bone lamina to the buccal bone wall immediately after tooth extraction, without the use of particulate grafting material. The study population consisted of a consecutive series of patients treated under routine clinical conditions.

The study was conducted in accordance with the ethical principles of the Declaration of Helsinki. Ethical approval for the retrospective analysis was granted by the Ethics Committee of the Universidad de Ciencias Médicas de La Habana, Facultad de Estomatología, La Habana, Cuba, on 20 November 2025 (protocol no. 2025/10). In accordance with the Ethics Committee approval, potentially eligible patients were contacted during their regular clinical follow-up, informed about the retrospective inclusion of their cases in the study, and asked to provide written informed consent for the use of their clinical and radiographic records for research purposes. All included patients provided this study-specific informed consent. Patients had also previously provided informed consent for the clinical procedures originally performed as part of routine care.

This retrospective observational study evaluated consecutive surgical cases treated between 7 September 2023 and 2 September 2025. Owing to the retrospective observational design, no randomization, treatment allocation, blinding procedures, or prospective trial registration were applicable. The study is reported in accordance with the STROBE recommendations for observational research.

### 2.2. Study Size

The study population was derived from the clinical records of 70 consecutive patients who underwent alveolar ridge preservation with the subperiosteal cortical lamina technique during the predefined study interval. Case inclusion was based on the availability of complete clinical documentation and the required radiographic follow-up for outcome assessment. Because the investigation was designed as a retrospective observational study based on routinely treated cases, no formal a priori sample size calculation was carried out. The final study size therefore corresponded to the total number of consecutive eligible cases available for analysis within the selected period.

### 2.3. Case Selection, Eligibility Criteria, and Outcome Assessment

This retrospective study included consecutively treated patients who had undergone single-tooth extraction with planned delayed implant placement and had been treated with a subperiosteal cortical lamina according to the routine clinical protocol of the center. The indication for this treatment had been established at the time of treatment on the basis of predefined clinical criteria routinely adopted in daily practice, whereas the present study retrospectively evaluated the corresponding clinical and CBCT records. Only patients who subsequently provided written informed consent for the retrospective use of their clinical and radiographic records were included in the final study analysis.

According to the routine clinical protocol, treatment with the cortical lamina was not performed in the presence of heavy smoking (more than 10 cigarettes per day), heavy alcohol intake, pregnancy or breastfeeding, uncontrolled systemic disorders potentially affecting healing, active or chronic periodontal disease requiring extraction, local periodontal conditions judged unsuitable for the planned cortical lamina approach, or the use of medications known to interfere with bone or soft tissue repair, including phenytoin, cyclosporine, dihydropyridine calcium channel blockers, anticoagulants, and bisphosphonates. Patients with a history of drug abuse, poor compliance, or psychological conditions potentially affecting adherence were also not considered suitable for this treatment approach.

In addition, patients who had received the same treatment but had already been enrolled in a previous clinical study, including the earlier randomized controlled trial [20], were excluded from the present analysis to avoid overlap between study populations.

The clinical indication for tooth extraction was retrieved from the patients’ records and classified into four categories: root fracture, root decay, endodontic failure, and crown fracture. These diagnostic categories were recorded descriptively to characterize the clinical conditions leading to extraction and were not used as allocation or subgrouping variables.

The presence of fistulas, dehiscences, perforations, or apical radiolucencies did not automatically preclude treatment and was evaluated from the available clinical and radiographic records when documented. Suitability for the cortical lamina approach was determined clinically at the time of surgery according to the extent of the defect and the feasibility of managing the site within the planned operative protocol. Sockets with limited defects that could be managed with this technique were treated accordingly, whereas sites with extensive buccal breakdown or lesions requiring additional regenerative procedures were not treated with the cortical lamina and, therefore, were not represented in the study sample.

All interventions had been performed by two experienced surgeons following the same operative protocol. Following confirmation of eligibility and acquisition of study-specific informed consent, clinical and CBCT data were subsequently retrieved from the records and entered into the study database for analysis. When applicable, radiographic measurements were performed according to predefined anatomical reference points and standardized assessment criteria.

### 2.4. Biomaterial Used

OsteoBiol^®^ Lamina^®^ Soft (Tecnoss^®^, Giaveno, Italy) is a collagenic bone lamina made of cortical bone of porcine origin that undergoes a process of superficial semi-decalcification, acquiring an elastic consistency while nevertheless maintaining the typical compactness of the bone tissue from which it originates.

### 2.5. Radiographic Acquisition

CBCT scans were obtained at two time points, before tooth extraction and immediately before re-entry for implant placement. All scans were performed with the same cone beam system (3D Hyperion MyRay X5, Cefla, Bologna, Italy), using a 10 × 8 cm field of view and a voxel resolution of 0.3 mm. The exposure settings were automatically determined by the unit (90 kVp, 15 mAs). Image evaluation was carried out on coronal sections at ×200 magnification with the manufacturer’s dedicated software (iRYS V10, Cefla, Bologna, Italy).

To improve consistency throughout the study period, the CBCT device underwent periodic calibration procedures according to the manufacturer’s instructions aimed at maintaining stable image acquisition and reducing the risk of dimensional inaccuracies.

### 2.6. Clinical Procedures

A single preoperative dose of 2 g amoxicillin combined with clavulanic acid was administered 2 h before surgery. Before the procedure, patients rinsed with 0.2% chlorhexidine, and paracetamol was given for immediate postoperative pain control. After local infiltration anesthesia, the selected tooth was approached surgically.

An intrasulcular incision was made with a scalpel, extending to the adjacent mesial and distal aspects. The buccal gingival margin was then carefully reflected with a periosteal elevator in order to expose the marginal portion of the alveolar crest. To facilitate atraumatic extraction, a thin, sharp, rounded chisel (mini beaver blade) was inserted into the sulcus along the periodontal ligament space, and gentle controlled movements were used to detach the most coronal periodontal fibers while minimizing the risk of injury to the buccal bone wall. The tooth was subsequently removed with forceps, and the socket was meticulously debrided.

**Figure 1:**
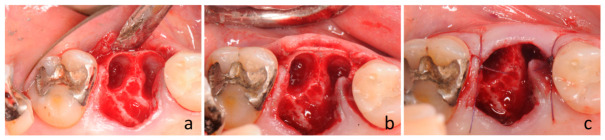
Clinical procedures after extraction of an upper molar. (**a**) Creation of a full-thickness envelope using a periosteal elevator; (**b**) cortical lamina placed between the bone plate and the periosteum; (**c**) suturing of the surgical site.

After extraction, a subperiosteal envelope was prepared on the buccal aspect of the socket by gently advancing a periosteal elevator between the periosteum and the underlying bone (Figure 1a). The dissection extended approximately 8–10 mm apically, while mesial and distal extension was restricted to the neighboring papillae so as to preserve soft tissue continuity.

A dried cortical lamina was then trimmed and shaped to fit the exposed buccal alveolar surface and inserted into the prepared subperiosteal envelope (Figure 1b). Any excess portion was removed, and the soft tissues were stabilized with interrupted sutures placed at the mesial and distal papillae, intentionally leaving the socket entrance uncovered (Figure 1c). Occasionally, a small coronal portion of the lamina remained exposed above the mucosal margin. This condition was considered clinically acceptable, as it did not appear to interfere with healing and was not associated with subsequent exposure-related complications. At the end of the procedure, light compression was applied with gauze soaked in sterile saline for approximately 5 min in order to stabilize the blood clot.

Fixation pins were employed when passive adaptation alone did not provide sufficient primary stability, particularly at sockets with buccal wall deficiencies, and in case of a curved buccal bone profile, aiming to adapt the cortical lamina to the outer contour of the buccal bone wall. The use of pins was regarded as an adjunctive stabilization measure rather than a deviation from the planned surgical approach.

**Figure 2:**
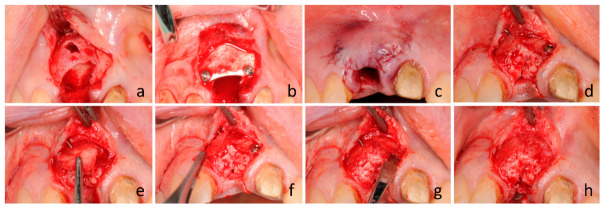
Clinical case. (**a**) Flap elevation including releasing incision after extraction of element 1.1; note the fenestration at the buccal bone; (**b**) the cortical lamina was placed on the buccal bone wall and fixed with pins; (**c**) closure of the wound with sutures; (**d**) re-entry after 6 months; note the presence of residues of the cortical lamina and pins; (**e**,**f**) removal of a residue slightly adhering to the subjacent bone; (**g**) use of a scalpel to detach the cortical lamina from the bone; (**h**) view of the bone healing.

These procedures had most often required releasing incisions (Figure 2a–c).

In selected cases with lingual wall defects, an additional cortical lamina was positioned on the lingual side.

### 2.7. Postoperative Care and Follow-Up

After surgery, patients were instructed to rinse with 0.2% chlorhexidine digluconate three times per day and to follow a modified toothbrushing regimen, avoiding mechanical cleaning of the treated area during the initial healing phase. Sutures were removed after 14 days, and a clinical examination was performed by the surgeon at the same appointment. During this visit, professional plaque control was also provided by a dental hygienist, who reinforced the oral hygiene instructions. A further supportive hygiene session was scheduled 8 weeks after surgery.

In cases involving esthetic areas, when clinically indicated, a provisional acrylic restoration (e.g., a Maryland bridge) was delivered on the day of surgery. Patients were then re-evaluated after a healing period of approximately 6 months for implant placement. A follow-up CBCT examination was obtained about 1 week before implant installation.

### 2.8. Tomographic Examinations

All CBCT measurements were performed by a single calibrated examiner (T.M.) according to a predefined measurement protocol. Calibration was established through repeated measurement sessions with senior supervision (D.B.) to verify landmark identification and procedural consistency. Prior to the final radiographic analysis, intra-examiner calibration confirmed excellent reproducibility (ICC > 0.9).

The procedure used to identify the corresponding cross-sectional image at T1 and T2 was adapted from the CBCT measurement protocol previously described by Mainetti et al. [20]. At T1, the cross-sectional image was selected on the panoramic reconstruction at the central position of the tooth/root to be extracted. The mesio-distal position of this section was recorded in relation to the neighboring teeth, which were used as stable anatomical references. At T2, after healing of the extraction site, the corresponding section was re-identified on the panoramic reconstruction using the same adjacent teeth and the previously recorded distances. The cross-sectional image was then oriented perpendicular to the alveolar ridge, and the same measurement protocol was repeated. This procedure was adopted to reproduce the same extraction-site position before and after healing, despite the anatomical changes caused by tooth extraction and ridge remodeling.

**Figure 3:**
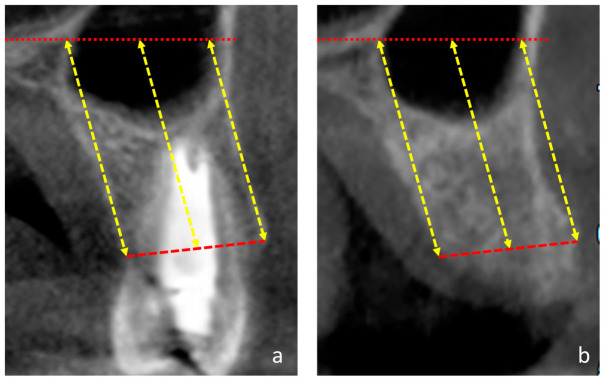
Cone beam computed tomography (CBCT) scans obtained (**a**) before and (**b**) approximately 6 months after tooth extraction. One line was drawn parallel to the nasal floor (red dotted line), and another connecting the buccal and lingual crestal bone peaks (red dashed line), representing the crestal bone width. The distance between the two lines was measured, parallel to the bone crest axis, at the buccal, central, and lingual aspects (yellow dashed arrows).

The buccal and lingual bone crests, as identified on the CBCT images, were used as the main coronal reference points. A line connecting these two landmarks was drawn and defined as the bucco-lingual line, corresponding to the width of the alveolar crest (Figure 3a,b).

In the maxilla, the floor of the nasal cavity was used as the reference plane. Measurements were performed on the cross-sectional image passing through the central axis of the alveolar process at T1 and through the center of the healed alveolar crest at T2. The vertical distances from the nasal floor to the buccal and lingual bone crests were recorded. In addition, the distance between the nasal floor and the midpoint of the BL line was measured.

In the mandible, the same measurement approach was adopted, using the basal mandibular border as the reference plane.

### 2.9. Statistical Analysis

The primary outcome was the horizontal dimensional change in the alveolar ridge, expressed as the difference in crestal width between baseline (T1) and follow-up (T2). The vertical change at the central aspect, defined as the distance between the reference plane (nasal floor in the maxilla or basal mandibular border in the mandible) and the midpoint of the BL line, was considered the key secondary outcome. All remaining measurements were regarded as exploratory and complementary.

For each variable, the distribution of the paired differences (T2–T1) was assessed using the Shapiro–Wilk test. When the distribution of the within-subject differences was compatible with normality, changes over time were analyzed using a paired *t*-test; otherwise, the Wilcoxon signed-rank test was applied. All tests were two-tailed, and statistical significance was set at α = 0.05. Statistical analyses were performed using GraphPad Prism (version 11.0.0 for Windows, GraphPad Software, Boston, MA, USA). Because the study was based on repeated measurements obtained from the same patients, all inferential analyses were conducted using paired statistical methods based on within-subject changes.

In addition to *p*-values, effect size estimates were calculated for the primary outcome and for the key secondary vertical measurement to quantify the magnitude of the observed changes. For paired comparisons, standardized mean-change effect sizes with small-sample correction (Hedges’ g for dependent samples) were derived from the paired differences. Effect sizes were interpreted according to conventional benchmarks (0.2, 0.5, and 0.8 for small, medium, and large effects, respectively), while also considering their potential clinical relevance.

No formal adjustment for multiple comparisons was applied. This choice was based on the prespecification of a single primary outcome, the identification of the central vertical measurement as a key secondary variable, and the exploratory nature of the remaining analyses. Accordingly, results from non-primary outcomes were interpreted as supportive and hypothesis-generating rather than confirmatory.

In addition to the overall analysis of the full sample, an exploratory subgroup analysis was conducted according to tooth region. Extraction sites were classified as anterior teeth (incisors and canines), premolars, or molars. This stratification was introduced because previous controlled data had mainly involved anterior and premolar sites, whereas the present retrospective series also included molars. The subgroup analysis was therefore intended to explore whether the dimensional changes observed with the cortical lamina approach remained similarly limited in molar regions. These assessments were considered ancillary and hypothesis-generating rather than part of the primary inferential framework.

## 3. Results

### 3.1. Study Population and Site Distribution

A total of 70 patients contributing 70 extraction sites were included in the study. The clinical indications for tooth extraction were root fracture in 21 sites, root decay in 20 sites, endodontic failure in 28 sites, and crown fracture in 1 site.

The study population comprised 27 men and 43 women, with a mean age of 63.7 ± 12.2 years. Overall, 49 sites were located in the upper jaw and 21 in the lower jaw (Table 1).

Nine sites presented buccal bone wall loss to varying degrees; in five of these sites, the loss was almost half of the depth of the extraction socket. In nine cases, fixation pins were used to ensure adequate primary stability and adaptation of the cortical lamina to the buccal bone contour. No complications were observed in any case, including those presenting an initial small exposure of the cortical lamina beyond the mucosal margin. Implants were inserted in all treated sites.

For exploratory purposes, the sites were stratified according to tooth region into incisors/canines (*n* = 21), premolars (*n* = 33), and molars (*n* = 16).

### 3.2. Tomographic Evaluations

The mean follow-up period, based on CBCT dates, was 6.1 ± 1.4 months.

In the overall sample, the mean crestal width decreased from 8.4 ± 2.8 mm before extraction to 7.9 ± 1.8 mm at follow-up, corresponding to a mean reduction of −0.6 ± 2.0 mm (Table 2). This change was statistically significant (*p* = 0.001).

The mean buccal height was 16.5 ± 6.4 mm at baseline and 16.6 ± 6.8 mm at follow-up, with a mean variation of 0.1 ± 2.3 mm, showing no statistically significant difference (*p* = 0.939).

The mean central height increased from 17.4 ± 6.2 mm before extraction to 17.8 ± 6.7 mm at follow-up, corresponding to a mean change of 0.4 ± 2.6 mm. This difference was not statistically significant (*p* = 0.531).

The mean lingual height decreased from 18.9 ± 6.6 mm at baseline to 18.3 ± 6.6 mm at follow-up, corresponding to a mean reduction of −0.6 ± 1.7 mm. This change was statistically significant (*p* = 0.005).

The standardized mean-change effect size for crestal width was −0.24, consistent with a small effect. For the central vertical measurement, the corresponding effect size was 0.15, suggesting a negligible-to-small effect.

### 3.3. Exploratory Analysis According to Tooth Region

As an exploratory analysis, extraction sites were also stratified according to tooth region in order to assess whether the dimensional changes observed in the overall sample were maintained across anterior, premolar, and molar sites (Table 3). When the extraction sites were stratified according to tooth region, the mean horizontal change was −0.3 ± 1.6 mm in incisors/canines, 0.1 ± 2.0 mm in premolars, and −2.2 ± 1.6 mm in molars. A statistically significant reduction in crestal width was observed only in molar sites (*p* < 0.01).

The mean central height changes were 0.5 ± 2.0 mm in incisors/canines, 0.7 ± 2.9 mm in premolars, and −0.4 ± 2.2 mm in molars, with no statistically significant differences within the individual subgroups.

For buccal height, the mean changes were 0.3 ± 1.8 mm in incisors/canines, 0.5 ± 2.6 mm in premolars, and −1.1 ± 1.8 mm in molars. A statistically significant reduction was found only in the molar subgroup (*p* < 0.05).

For lingual height, the mean changes were −0.6 ± 2.0 mm in incisors/canines, −0.4 ± 1.2 mm in premolars, and −1.0 ± 1.8 mm in molars. No statistically significant differences were observed within the subgroups.

In summary, molar sites showed greater mean dimensional reductions than anterior and premolar sites, particularly for crestal width and buccal height.

## 4. Discussion

The present retrospective clinical study evaluated the dimensional changes in extraction sites treated with a subperiosteal heterologous cortical collagenic bone lamina under routine clinical conditions. Overall, the findings showed limited post-extraction dimensional changes, with a mean crestal width reduction of −0.6 mm and no significant change at the central vertical aspect. These observations suggest that the cortical lamina approach may be clinically useful as a strategy for alveolar ridge preservation without the use of particulate grafting material in daily practice. However, because of the retrospective design and the absence of a parallel control group, the present findings should be interpreted as associative clinical observations rather than as definitive evidence of efficacy.

The present results are consistent with those observed in our previous randomized controlled trial, in which the same general approach was associated with minimal dimensional changes in the test group and significantly better ridge preservation than unassisted healing [20]. In that controlled setting, horizontal and vertical changes in the G-ARP group were negligible, whereas the untreated control sites showed marked reduction in both ridge width and height. Taken together, the two studies suggest that the protective effect associated with subperiosteal cortical lamina placement is not limited to an experimental controlled setting but can also be observed in a broader retrospective series derived from routine care.

At the same time, the comparison with the previous RCT also helps to explain an important difference in the present findings. In the randomized trial, only anterior and premolar teeth were included, whereas molar sites were excluded by design.

In the current retrospective series, molars were also included in order to extend the clinical applicability of the protocol beyond the anatomical spectrum covered by the previous trial. This distinction is relevant because the exploratory subgroup analysis suggested that the overall dimensional reduction observed in the present study was influenced mainly by the molar subgroup, in which the reduction in crestal width was more pronounced and buccal height also decreased significantly. However, this finding should be interpreted cautiously because the molar subgroup included a limited number of sites. Therefore, the observed trend should be considered hypothesis-generating rather than definitive evidence of a region-specific difference in the effectiveness of the technique.

This finding is not necessarily inconsistent with the previous RCT [20]. On the contrary, it is coherent with one of the main limitations already acknowledged in that trial, namely that the results primarily applied to anterior and premolar sites and should be extrapolated to molar regions with caution. Nevertheless, bone width loss in molar regions was in the present study limited to 18.5%, a reduction that still appeared compatible with implant placement.

Several anatomical and biomechanical factors may contribute to this pattern. Molar extraction sites are often characterized by wider sockets, more complex root anatomy, and a broader alveolar envelope, all of which may increase the magnitude of remodeling after extraction. In addition, in the present series a relevant proportion of molar sites were located in the upper jaw, where the palatal/lingual component may have contributed to the observed dimensional changes. Within this context, the less favorable behavior of molar sites should not be interpreted as a failure of the technique, but rather as an indication that the response may be site-dependent and that posterior regions may represent a more demanding clinical indication. These findings suggest that, in molar regions, the use of alternative strategies or technical modifications of the G-ARP may deserve consideration in order to improve dimensional stability. Although favorable results obtained with a buccal cortical lamina in molar sites have been described [12], the available evidence is still limited. In particular, the published case report involved only four molars, only one of which was located in the upper jaw, within an overall series of nine cases. The present study could help to fill this gap.

The concept underlying the G-ARP technique is consistent with the principles of Guided Tissue Regeneration (GTR), first introduced by Sture Nyman, in which selective tissue isolation allows the regeneration of a specific tissue type [21,22,23]. In this sense, the present approach is intended to create a healing environment more favorable to alveolar bone preservation and regeneration by limiting unwanted soft-tissue interference, in analogy with the protection of the periodontal ligament in GTR procedures. At the same time, as already discussed in the previous RCT, the mechanism responsible for the apparent ridge-preserving effect of the cortical lamina is likely multifactorial. The available clinical data are consistent with a predominantly mechanical contribution, whereby the lamina may act as a space-maintaining and protective barrier for the buccal bone wall during the early healing phase, thereby limiting soft-tissue collapse and reducing external pressure on the healing compartment.

Although concepts such as periosteal inhibition have been proposed, direct biological evidence remains lacking, and the clinical effect is more appropriately interpreted within a broader mechanical-biological framework. The present retrospective findings do not resolve this mechanistic issue, but they remain consistent with the same interpretative model. A conceptually related mechanical effect may also be achieved through non-grafting approaches based on immediate closure or sealing of the extraction socket by means of prosthetic or restorative components. In this context, provisional restorations, immediate implant-supported temporaries, and customized healing abutments have been proposed as strategies capable of stabilizing the blood clot, supporting the peri-implant soft tissues, and reducing early collapse of the alveolar profile [24,25,26,27,28,29,30]. Although their effect is indirect and mainly mediated by mechanical support of the periosteal-mucosal envelope, these observations further support the view that early dimensional stability may depend, at least in part, on mechanical protection of the healing compartment rather than exclusively on the use of grafting materials.

A conceptually related, although biologically distinct, approach is represented by the socket-shield technique, first described by Markus Hürzeler et al., in which a buccal root fragment is intentionally retained in situ to preserve the contour of the alveolar ridge [31]. Unlike socket-shield procedures, the present G-ARP approach does not rely on the preservation of dental tissues, but rather on the creation of a mechanically protected and space-maintained environment, thereby reducing dependence on more technique-sensitive biological assumptions.

From a clinical standpoint, the limited dimensional changes observed in the overall sample suggest that alveolar ridge preservation performed without particulate grafting material may help maintain a ridge contour suitable for delayed implant placement, potentially reducing the need for secondary regenerative procedures. This practical implication is in line with the conclusion of the previous RCT, in which the short-term preservation of ridge morphology was considered potentially advantageous during the pre-implant healing phase. However, the present subgroup findings indicate that this benefit may be less predictable in molar regions, where remodeling appears more pronounced.

Another relevant aspect is the resorbable nature of the cortical lamina. This does not eliminate the need for a subsequent surgical intervention when a delayed implant placement protocol is planned, as in the present study. Rather, the potential advantage is that the cortical lamina itself does not require a dedicated second procedure for barrier removal, as would be required with a non-resorbable d-PTFE membrane. However, this advantage should be interpreted with caution, because in selected cases fixation pins may be required to obtain adequate primary stability or adaptation of the lamina to the buccal bone contour. In such situations, a re-entry procedure may still be necessary for pin removal, although this need is related to the fixation device rather than to the cortical lamina itself.

In this perspective, the present investigation should be considered a first clinical step in the evaluation of this graft-free cortical lamina approach using a delayed implant placement protocol. A subsequent development of this technique may involve its application in association with immediate implant placement, where the resorbable nature of the lamina could be particularly advantageous by avoiding an additional intervention specifically related to barrier removal.

However, the resorption pattern of the cortical lamina remains incompletely characterized. Although the material is considered resorbable, complete disappearance has not yet been clearly documented. Available experimental data indicate progressive degradation rather than rapid elimination, with residual portions still detectable during healing and, in some instances, incorporated into newly formed bone [32,33]. For exploratory purposes, we report a case at which, at the time of implant installation after about 6 months of healing, the cortical lamina was partially resorbed and presented a small region apparently integrated into the underlying bone wall (Figure 2d–h).

Generally, the cortical lamina was stable when placed into the envelope prepared on the buccal bone wall of the extraction socket. However, when the primary stability of the cortical lamina was not guaranteed or its adaptation to the buccal bone wall was not adequate, pins were used to overcome the problem (Figure 2b). This technique obviously requires a re-entry procedure.

The present study has several limitations that should be acknowledged. First, its retrospective observational design does not provide the same level of methodological control as the previous randomized trial. Unlike the RCT discussed above, which included a control arm and blinded outcome assessment, the current investigation reflects a consecutive clinical series without a parallel untreated comparison group. Second, the follow-up period was relatively short, being limited to approximately 6 months. Third, the analysis was based exclusively on CBCT measurements and therefore does not provide histological information on the biological quality of the regenerated tissue, a limitation already noted in the previous trial as well. In addition, the spatial resolution of the CBCT system used in the present study should be taken into account. The voxel size was 0.3 mm, and therefore small linear differences close to this dimension may fall within the intrinsic measurement uncertainty of the imaging method. For this reason, minor dimensional variations, particularly those below or close to 0.5 mm, should not be overinterpreted as exact absolute changes at the submillimetric level. Greater emphasis should instead be placed on the overall dimensional trends and on changes clearly exceeding the expected range of radiographic measurement variability.

In addition, although the corresponding T1 and T2 sections were identified using the neighboring teeth and the recorded mesio-distal position on the panoramic reconstruction [20], exact three-dimensional image registration was not performed. Therefore, minor residual variability in section matching cannot be completely excluded. Nevertheless, the use of adjacent teeth as stable anatomical references, standardized perpendicular cross-sectional orientation, and examiner calibration was intended to improve the reproducibility and reliability of the radiographic assessment.

Baseline heterogeneity should also be taken into account, since 9 sites presented some degree of initial buccal bone wall loss, including 5 sites in which the defect involved almost half of the socket depth. This feature may have influenced the dimensional outcomes, as sites with a compromised buccal wall may also undergo partial width recovery during healing, thereby affecting the overall mean changes. Finally, the subgroup analysis according to tooth region was exploratory and should not be interpreted as definitive evidence of regional differences, particularly in view of the limited number of molar sites. Despite these limitations, the present study provides a clinically relevant indication of the practical boundaries within which the technique may be applicable under real-world conditions.

While the above-discussed randomized trial [20] documented the behavior of the cortical lamina approach under controlled conditions in anterior and premolar sites, the present retrospective series broadens the clinical perspective by evaluating the same treatment concept in a more heterogeneous population that also included molars. The present study suggests that subperiosteal cortical lamina placement may represent a useful approach for alveolar ridge preservation without particulate grafting material, while also indicating that site-related anatomical factors, particularly in molar regions, may influence the final dimensional outcome.

Nevertheless, further studies are needed to confirm the present findings. Future research should investigate the healing fate of the cortical lamina, including its possible resorption, integration, or long-term persistence, and should determine whether incomplete resorption may lead to complications or indicate the need for surgical removal. In addition, the quality of the newly formed bone, the possible need for adjunctive grafting procedures, and the potential application of cortical lamina in the treatment of bone defects deserve further investigation.

## 5. Conclusions

Within the limits of this retrospective uncontrolled case series, the subperiosteal cortical lamina approach was associated with limited post-extraction dimensional changes under routine clinical conditions. In the overall sample, horizontal ridge reduction was small, while the central vertical dimension remained substantially stable. Exploratory subgroup analysis suggested less favorable dimensional stability at molar sites; however, because of the limited number of molar sites, this finding should be interpreted cautiously and considered hypothesis-generating rather than definitive evidence of a region-specific effect. Overall, these findings should be interpreted as supportive observational evidence rather than definitive proof of efficacy. Further controlled studies are needed to confirm the clinical effectiveness of this technique and to better define its indications, particularly in molar regions.

## Figures and Tables

**Table 1:** Demographic characteristics of the study population and distribution of the 70 extraction sites according to tooth region and jaw. Age is expressed as mean (standard deviation). # = number of cases.

	Total (#)	Male (#)	Female (#)	Age (Years)	Upper (#)	Lower (#)
Total	70	27	43	63.7 (12.2)	49	21
Incisors/Canines	21	6	15	66.7 (14.0)	17	4
Premolars	33	11	22	64.5 (11.1)	20	13
Molars	16	10	6	58.4 (10.9)	12	4

**Table 2:** Tomographic measurements before tooth extraction and at follow-up in the overall sample (*n* = 70). Data are expressed as mean ± standard deviation. Differences represent post-extraction minus pre-extraction values (** *p* < 0.01). *p*-values refer to within-site comparisons over time and were obtained using paired *t*-test or Wilcoxon signed-rank test, as appropriate based on normality of paired differences.

Data in Millimeters	Width	Height		
		Buccal	Central	Lingual
Pre-extraction	8.4 ± 2.8	16.5 ± 6.4	17.4 ± 6.2	18.9 ± 6.6
Post-extraction	7.9 ± 1.8	16.6 ± 6.8	17.8 ± 6.7	18.3 ± 6.6
Difference	−0.6 ± 2.0 **	0.1 ± 2.3	0.4 ± 2.6	−0.6 ± 1.7 **
*p*-values	0.001	0.939	0.531	0.005

**Table 3:** Changes in tomographic measurements according to tooth region. Data are expressed in millimeters as mean ± standard deviation and represent post-extraction minus pre-extraction values. Asterisks indicate statistically significant within-group changes over time (* *p* < 0.05; ** *p* < 0.01). *p*-values were obtained using paired *t*-test or Wilcoxon signed-rank test, as appropriate based on normality of paired differences.

	Width	Height		
		Buccal	Central	Lingual
Total	−0.6 ± 2.0 **	0.1 ± 2.3	0.4 ± 2.6	−0.6 ± 1.7 **
Incisors/Canines	−0.3 ± 1.6	0.3 ± 1.8	0.5 ± 2.0	−0.6 ± 2.0
Premolars	0.1 ± 2.0	0.5 ± 2.6	0.7 ± 2.9	−0.4 ± 1.2
Molars	−2.2 ± 1.6 **	−1.1 ± 1.8 *	−0.4 ± 2.2	−1.0 ± 1.8

## Data Availability

The raw data supporting the conclusions of this article will be made available by the authors on request.
